# La dentisterie holistique au service de la santé globale

**DOI:** 10.11604/pamj.2018.30.102.12713

**Published:** 2018-06-06

**Authors:** Saida El Khayati, Amal El Yamani

**Affiliations:** 1 Service de Prothèse Conjointe du Centre de Consultation et de Traitement Dentaire de Rabat, Maroc

**Keywords:** Amalgames, intoxication, santé globale, Amalgams, intoxication, overall health

## Abstract

Les amalgames dentaires constituent des matériaux d'obturation utilisés pour le traitement des lésions carieuses depuis plus de 150 ans. Cependant le mercure servant de base à la préparation des amalgames est universellement considéré comme un toxique très dangereux pour l'être humain. L'objectif de ce travail est de monter à travers un cas clinique certains signes locaux et généraux d'intoxication au mercure, les précautions à prendre lors de la dépose des amalgames, ainsi que les solutions de remplacements actuels qu'on doit proposer à nos patients. En réalité la toxicité des amalgames n'est que la partie émergée de l'iceberg. Les autres métaux employés en dentisterie peuvent également provoquer des problèmes importants en induisant des effets galvaniques, des intolérances, des allergies, des réactions d'intoxication ou de sub-intoxication. En matière de santé publique, ces motifs justifient amplement l'intérêt que tout thérapeute doit accorder aux alternatives prothétiques n'incluant pas de métal.

Dental amalgams are dental filling materials which have been used to fill cavities caused by tooth decay for more than 150 years. However, mercury used as a basis for the preparation of amalgams is universally regarded as toxic and thus very dangerous for the human being. This clinical case report aimed to describe some local and general signs of mercury poisoning, to emphasize necessary precautions during amalgam filling and to highlight current replacement techniques that should be proposed to our patients. In effect, amalgam toxicity is just the tip of the iceberg. Other metals used in dentistry can cause significant problems bringing about galvanic effects, intolerances, allergies, toxic and subtoxic reactions. In public health, this is a sufficient reason to justify therapists’ interest in non-metal prosthetic alternatives.

## Introduction

Depuis la révolution industrielle de la fin du 19^ème^ siècle, nous avons tous été exposés à un univers de plus en plus toxique et pollué. Parmi les plus dangereux de ces polluants figure le mercure considéré comme le métal lourd non radioactif le plus toxique. Il n'existe aucune fonction métabolique pour laquelle on sache que le mercure serait indispensable. Le mercure est considéré comme toxique quelle que soit sa concentration dans l'organisme et peut causer une très grande variété de perturbations [1]. Les amalgames dentaires constituent des matériaux d'obturation utilisés pour le traitement des lésions carieuses depuis plus de 150 ans. Il s'agit de dispositifs médicaux dont l'efficacité thérapeutique (et, en particulier, l'action bactéricide) est démontrée [2]. Cependant le mercure servant de base à la préparation des amalgames est universellement considéré comme un toxique très dangereux pour l'être humain. La dispersion des résidus d'amalgames dans l'environnement doit être soigneusement évitée car le mercure en se répandant, pollue les nappes phréatiques, les terres et les rivières de façon irréversible [3]. La présence de mercure dans la bouche sous forme d'amalgames n'est pas souhaitable, donc il serait plus sage de déposer les amalgames de nos patients surtout en présence de signe d'intoxication. Lors de la dépose des précautions méticuleuses doivent être prise afin de ne pas augmenter de façon dramatique la charge en mercure du patient. Une fois déposé, cette obturation noire devra être remplacée par une restauration dont les caractéristiques permettent de réaligner l'art dentaire avec notre époque en tenant compte de l'impact de sommation des agressions sur la vitalité pulpaire. Les inlays-onlays esthétiques respectent les exigences de la dentisterie moderne que sont la préservation tissulaire, la biomécanique, l'étanchéité et la biocompatibilité. Il s'agit alors d'une thérapeutique qui concilie les quatres impératifs (biologiques, mécaniques, fonctionnels et esthétique) du puzzle physiologique de Magne et Belser [4]. L'objectif de ce travail est d'illustrer à travers un cas clinique le service que peut rendre la dentisterie holistique à la santé globale de nos patients.

## Patient et observation

**Figure 1:**
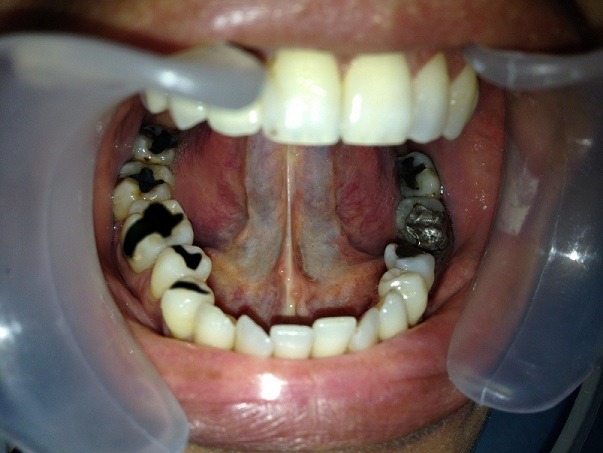
L’arcade inférieure avant mise en état

**Figure 2:**
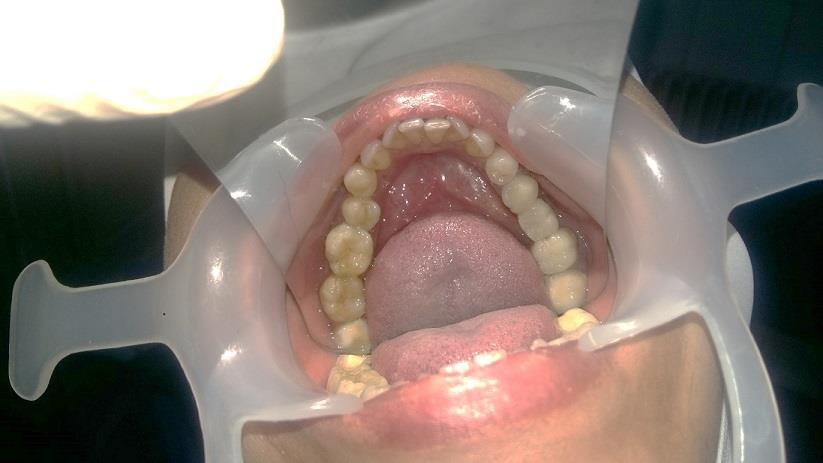
Résultat final de la réhabilitation de l’arcade inférieure

Dans le cadre de l'activité hospitalière du centre de consultation et de traitement dentaire de Rabat, nous avons reçu Madame X âgée de 37 ans pour une réhabilitation esthétique fixée. L'anamnèse a révélé un syndrome de fatigue chronique inexpliqué et une rhinite allergique qui remonte à six ans. La patiente est adressée par son médecin traitant pour des aphtes à répétition, picotements et brulures au niveau de la langue. L'examen exobuccal a objectivé un visage ovalaire symétrique, des étages faciaux égaux, un profil convexe et une légère prognathie maxillaire. La palpation des muscles et des aires ganglionnaires est asymptomatique. L'examen des articulations temporo-mandibulaire est sans particularité, bien que son ouverture buccale limitée à deux doigt et demi décrit un trajet en baïonnette. L'examen endobuccal a mis en évidence des tatouages gingivaux diffus, une hygiène bucco-dentaire moyenne avec l'indice de plaque de Silness et Loe quantifié à un et l'indice gingival estimé à deux degrés. A l'examen dentaire, la patiente a présenté des absences dentaires (18, 17, 16, 28, 36), des caries (11, 38, 37, 35, 44, 45, 46, 47, 48), un bridge céramo-métallique de la 24 à la 27, une couronne céramo-métallique au niveau de la 12 et surtout plusieurs obturations coronaires à l'amalgame (38, 37, 35, 44, 45, 46, 47, 48) (Figure 1). L'examen radiographique nous a renseigné sur l'étendue en profondeur des obturations à l'amalgame, ainsi que la qualité du traitement canalaire de la 36. Après consentement de la patiente on a décidé de déposer toutes les obturations coronaires à l'amalgame et on a opté pour un Bridge céramo-métallique trois éléments (37, 36, 35) et quatre onlays en composite de laboratoire (44, 45, 46, 47) (Figure 2). La dépose des amalgames s'est faite dent par dent à l'intervalle de 15 jours afin de permettre l'élimination progressive du mercure mais sans tenir compte d'une chronologie particulière. En effet, on ne dispose pas d'un multimètre digital pour identifier l'amalgame le plus actif qui devrait être déposé en premier. La protection du praticien et du patient est de règle; elle passe par le port de lunettes et de masque (de préférence en charbon), la pose obligatoire de la digue, l'utilisation d'une aspiration chirurgicale puissante et une irrigation abondante afin de refroidir l'amalgame et éviter son échauffement.

En effet le mercure est un métal liquide volatile qui s'évapore à partir d'un effet thermique au-delà de 40 degrés. L'amalgame a été découpé en fragments pour éviter une pulvérisation et ses conséquences. Cela est difficile pour les très petits amalgames mais possible grâce à des fraises à détourer très fines. Après la dépose on a prescrit du charbon actif sous forme de bain de bouche et de pastilles car par effet de pompe ce charbon va absorber d'éventuels débris de mercure qui seraient présents en bouche ou dans le tube digestif. Des conseils diététiques ont été donnés à la patiente pour favoriser l'élimination rénale et digestive des radicaux libres. Après avoir déposé tous les amalgames, on a entamé la réhabilitation prothétique proprement dite, pour cela on a procédé comme suit: préparation périphérique de la 35 et 37; empreinte pour inlay-core pour la 35 et 37; essayage, scellement des inlays cores; empreinte secondaire par wash technique; essayage de l'armature métallique. L'emploi du métal s'avère indispensable pour des raisons financiers, mais il est impératif de conserver le même alliage pour l'ensemble des travaux. Le nom et la composition exacte de l'alliage doivent être soigneusement consignés dans le dossier dentaire afin de permettre un suivi, même si le patient est amené à changer de dentiste. Montage sur articulateur: modèle supérieur au moyen de l'arc facial et le modèle inferieur secondaire par mordu occlusal; Choix de la couleur; essayage de la céramique à l'état de biscuit; scellement du bridge et de la CCM au CVI par adjonction de résine; taille pour onlay sur la 44, 45, 46 et 47 tout en préservant le verre ionomère comme fond de cavité. Lors de la préparation dentaire pour onlay on a conservé les crêtes et on a préservé la vitalité pulpaire, le fond de la cavité est plat et la préparation ne présente pas d'angles vifs, qui risqueraient de fracturer la pièce prothétique. Les parois ont une dépouille minimale de 10° et la limite périphérique est un épaulement à angle interne arrondi sans chanfrein associé. Pour éviter de fragiliser le joint, on a vérifié que les dents antagonistes n'ont pas de contacts occlusaux statiques ni dynamiques avec les limites de notre préparation (absence d'interférence). Provisoires réalisées par technique d'auto-meulage en maintenant un jet d'eau air continue au cours de la polymérisation de la résine peut ne pas causer des dommages menaçant la vitalité pulpaire; empreinte secondaire par Technique double mélange; essayage des onlays et collage Une fois les obturations à l'amalgame éliminées, les troubles ont commencé à s'estomper, avant de disparaître; la patiente a déclaré une nette amélioration de son état de son santé général ainsi les signes locaux en rapport avec le gout métallique, les sensations de picotements et de brulure de la langue ont disparu en faveur d'une sensation de bien-être.

## Discussion

Depuis quelques années la dentisterie holistique, parfois appelée énergétique, connaît un véritable engouement. La polémique sur les obturations à l'amalgame a permis de réaliser que la nature des métaux insérés en bouche peut altérer notre état de santé et contribuer à la genèse de maladies. Des recherches scientifiques ont démontré que la corrosion des amalgames dentaires par la mastication, l'exposition à l'oxygène de l'air respiré, les acides des aliments, la température et l'effet électrolytique des minéraux dans la salive (appelé galvanisation buccale) provoque une libération continuelle de vapeurs de mercure métallique dans l'organisme 24 heures sur 24. La consommation de mercure inorganique dans la salive avalée est 10 à 100 fois supérieure aux standards connus d'exposition [5]. Le mercure ainsi libéré dans la cavité buccale peut soit diffuser via les sinus vers les tissus. cérébraux et s'y accumuler, soit être inspiré dans les poumons et aboutir dans le système sanguin, soit passer dans le tube digestif où il sera partiellement méthylé ou transformé en sels (chlorure mercurique etc). Cette transformation (favorisée par certaines bactéries) va le rendre soluble et lui permet de pénétrer partiellement dans la circulation sanguine où il se fixe de manière très passagère sur les globules rouges. Il aboutira finalement aux reins et sera en partie excrété. Mais il pourra également se fixer dans le tissu rénal et l'intoxiquer de manière irréversible en créant des pathologies telles l'insuffisance rénale chronique [3]. Le Service de Santé Publique des Etats Unis a décrété que l'exposition chronique au mercure des amalgames dentaires n'est pas sans risque pour la population [6]. De plus, l'organisation Mondiale de la Santé a confirmé, en 1991, que l'amalgame dentaire est la plus importante source de vapeur de mercure dans les populations exposées non-industriellement, excédant significativement celle provenant des aliments ou de l'air. Mutter souligne que « la perfidie d'une intoxication réside dans le fait qu'il peut s'écouler jusqu'à quinze années ou plus, entre le début de l'intoxication et l'apparition des symptômes. (c'est le cas pour cette patiente), lorsque d'infimes quantités de mercure sont absorbées sur une longue durée, et s'accumulent dans l'organisme, comme c'est le cas pour les porteurs d'amalgames, il survient une intoxication insidieuse et chronique» [7].

Dans ces conditions, Le médecin aurait des difficultés extrêmes à relier des symptômes sub-cliniques à une toxicité du mercure (dans le cas de notre patiente le syndrome de fatigue chronique était considéré comme étant idiopathique alors que s'est probablement dû à une intoxication au mercure tenant compte de la relation cause à effet). Alfred Stock, un éminent chimiste allemand fit également allusion au problème de diagnostiquer la toxicité du mercure comme source première de symptômes cliniques dans les premiers stades d'empoisonnement systémique dès 1926 [8]. Le mercure est en particulier mis en cause dans la multiplication des « maladies émergentes », c'est-à-dire des maladies qui se multiplient depuis les années 1980: fibromyalgie, syndrome de fatigue chronique (SFC), allergies de toutes sortes, dépression, spasmophilie, troubles de la sensibilité neuro-musculaire, infections chroniques, mycoses récidivantes, troubles du métabolisme, troubles de l'attention et l'hyperactivité chez les enfants, migraines et douleurs diffuses. L'intoxication au mercure a été aussi incriminée dans la maladie de Parkinson, la sclérose en plaque, l'autisme et de nombreux cas de cancers [9]. La présence de mercure dans la bouche sous forme d'amalgames n'est pas souhaitable, mais la dépose doit être considérée comme une situation extrême pour l'organisme sur le plan toxicologique dans laquelle des précautions méticuleuses doivent être prises sinon à une atteinte toxicologique chronique va s'ajouter temporairement un épisode aigu dû à la dépose [3]. Il faut en outre s'abstenir chez la femme enceinte ou qui projette de l'être et chez celle qui allaite [2]. De plus, il est nécessaire de se donner les moyens d'une solution de remplacement de qualité. En cas de délabrement important, les restaurations directes sont inadaptées, mieux vaut recourir aux restaurations partielles indirectes, en privilégiant la céramique ou le composite de laboratoire pour éviter les phénomènes galvaniques liés au métal.

Mis à part la suppression de l'électro-galvanisme lié au métal, les restaurations esthétiques indirectes ou encore les inlays onlays esthétiques permettent de répondre aux exigences de la dentisterie moderne à savoir la préservation tissulaire, la biomécanique, l'étanchéité et la biocompatibilité. En effet l'avènement des nouvelles générations de résines composites de laboratoire permet de réaliser des restaurations dont la résistance et l'esthétique sont devenues remarquables. En parallèle, nous avons assisté à une révolution biologique qui nous permet aujourd'hui de réaliser un scellement étanche du complexe dentino-pulpaire garant de la biocompatibilité de nos restaurations. En réalité, beaucoup de praticien dans notre pays le Maroc sous estiment les dangers que représentent les amalgames. Pourtant, les études prouvant le contraire existent et ils sont aujourd'hui interdits dans plusieurs pays. Au Japon et en Russie les amalgames dentaires au mercure ont été abandonnés depuis plus de trente ans. La Norvège condamne l'usage du mercure dans toutes ses applications. En Suède, on ne pose plus d'amalgames depuis 1999. En Allemagne, la pose d'amalgames dentaires n'est même plus enseignée aux étudiants dans les facultés dentaires. Nous sommes à l'aube d'une révolution car la technologie offre enfin une alternative satisfaisante au métal. Profitons-en en faisant le choix de l'esthétique et de la biocompatibilité pour le plus grand bénéfice de la santé des patients. On a tendance à croire que le problème se limite au mercure. En réalité la toxicité des amalgames dentaires n'est que la partie émergée de l'iceberg. Les autres métaux employés en dentisterie (couronnes, bridges, implants, dentiers à châssis métallique, appareillages d'orthodontie, coiffes pour les dents de lait) peuvent également provoquer des problèmes importants en induisant des effets galvaniques, des intolérances, des allergies, des réactions d'intoxication ou de sub-intoxication (tels l'étain ou l'argent par exemple).

## Conclusion

En matière de santé publique, ces motifs justifient amplement l'intérêt que tout thérapeute doit accorder aux phénomènes galvaniques dans la cavité buccale. Chaque fois que possible, il faut privilégier les solutions prothétiques n'incluant pas de métal. Les progrès technologiques permettent aujourd'hui de réaliser des implants, des couronnes et des bridges en zircone, une céramique très solide, mieux tolérée que le métal. Ces prothèses, plus esthétiques que celles avec métal, doivent être privilégiées chaque fois que les conditions de réalisation le permettent. Si l'emploi du métal s'avère indispensable pour des raisons techniques ou économiques, il est impératif de conserver le même alliage pour l'ensemble des travaux. Le nom et la composition exacte de l'alliage doivent être soigneusement consignés dans le dossier dentaire afin de permettre un suivi. Par ailleurs, et concernant le domaine de la recherche, il est nécessaire de réaliser plus d'études à haut niveau de preuve scientifique en rapport avec la surveillance de l'intoxication mercurielle et la dépose des amalgames afin de standardiser les protocoles de prise en charge. C'est ici que la réalisation d'essais cliniques contrôlés et randomisés trouve tout son intérêt bien que ce type d'études demeure difficile.

## Conflits d’intérêts

Les auteurs ne déclarent aucun conflit d'intérêts.
